# Conversion Ratio between Botox^®^, Dysport^®^, and Xeomin^®^ in Clinical Practice

**DOI:** 10.3390/toxins8030065

**Published:** 2016-03-04

**Authors:** Francesco Scaglione

**Affiliations:** Department of Oncology and Onco-Hematology, University of Milan, Via Vanvitelli 32, 20129 Milan, Italy; francesco.scaglione@unimi.it; Tel.: +39-02-5031-6950; Fax: +39-02-5031-7100

**Keywords:** botulinum neurotoxin, onabotulinum toxin-A, abobotulinum toxin-A, incobotulinum toxin A

## Abstract

Botulinum neurotoxin has revolutionized the treatment of spasticity and is now administered worldwide. There are currently three leading botulinum neurotoxin type A products available in the Western Hemisphere: onabotulinum toxin-A (ONA) Botox^®^, abobotulinum toxin-A (ABO), Dysport^®^, and incobotulinum toxin A (INCO, Xeomin^®^). Although the efficacies are similar, there is an intense debate regarding the comparability of various preparations. Here we will address the clinical issues of potency and conversion ratios, as well as safety issues such as toxin spread and immunogenicity, to provide guidance for BoNT-A use in clinical practice. INCO was shown to be as effective as ONA with a comparable adverse event profile when a clinical conversion ratio of 1:1 was used. The available clinical and preclinical data suggest that a conversion ratio ABO:ONA of 3:1—or even lower—could be appropriate for treating spasticity, cervical dystonia, and blepharospasm or hemifacial spasm. A higher conversion ratio may lead to an overdosing of ABO. While uncommon, distant spread may occur; however, several factors other than the pharmaceutical preparation are thought to affect spread. Finally, whereas the three products have similar efficacy when properly dosed, ABO has a better cost-efficacy profile.

## 1. Introduction

Botulinum neurotoxin injections are a valuable treatment for many therapeutic indications and have revolutionized the treatment of spasticity and dystonia. Botulinum toxin is produced by anaerobic fermentation of the bacterium *Clostridium botulinum*. A number of different *C. botulinum* strains have been identified; they produce eight immunologically distinct serotypes (type A–H) and consist of the botulinum neurotoxin complexed with a number of associated proteins.

Neurotoxin type A preparations are the most widely used for therapeutic application. There are currently three leading botulinum neurotoxin type A (BoNT/A) products on the market in the Western Hemisphere: onabotulinumtoxinA (ONA; Botox/Vistabel, Allergan Inc., Irvine, CA, USA), abobotulinum toxin A (ABO; Dysport/Ipsen Limited, Slough Berkshire, UK), and incobotulinum toxin A (INCO; Xeomin/Bocouture, Merz Pharmaceuticals GmbH, Frankfurt, Germany).

In nature, BoNT-A is synthesized as macromolecular protein complexes [1]. These protein complexes are referred to as progenitor toxins and consist of nontoxic accessory proteins (NAPs) bonded to the 150-kD active neurotoxin. The BoNT-A progenitor toxins vary in molecular weight (300–900 kD) depending on the composition of NAPs and the manufacturing process [2]. The 150-kD neurotoxin must dissociate from NAPs to exert its pharmacologic effects. Dissociation occurs in physiologic pH conditions.

Although there are no clear differences in effectiveness between the various formulations, their comparability is intensely debated. The present study was not intended to be a systematic analysis of the effectiveness and safety of various preparations. Rather, we focused on their comparability. In particular, we examined the clinical issues of potency and conversion ratios, as well as safety issues such as toxin spread and immunogenicity, to provide information about BoNT-A use in clinical practice.

## 2. Potency

Although the various BoNT-A products differ in NAP composition, the 150-kD neurotoxin is the active moiety that ultimately inhibits acetylcholine release. Since the toxin moiety is the same in all pharmaceutical preparations, differences in potency depend of the amount of active toxin available. To become fully activated, the single chain 150-kD neurotoxin must be cleaved from the protein complex. All of the commercially available BoNT-A formulations are composed of the 150-kD neurotoxin with NAPs with the exception of INCO, which contains only the 150-kD neurotoxin. However, the manufacturing process may affect the amount of active toxin; for instance, enzymes added to increase the percentage of cleaved active toxin may denature the neurotoxic protein itself.

Therapeutically available BoNT-A formulations contain variable percentages of inactive toxin that contribute to the overall protein load without contributing to efficacy. For this reason, the potency is expressed in biological units. Potency is related to the quantity of toxin (in ng of protein content, *i.e.*, 150 kD neurotoxin including NAPs) required to achieve a median lethal dose (LD50) unit [3,4]. However, many factors affect the mouse LD50 bioassay including mouse strain, sex, age, volume and route of injection, time of examination after injection, and delivery vehicle or reconstituting buffer. Moreover, the LD50 units of BoNT products are not standardized across manufacturers. Due to the lack of LD50 bioassay harmonization, the unit potencies of BoNT formulations cannot easily be compared. For this reason, physicians should consider that although the active molecule is botulinum neurotoxin type A, different forms of the complex can affect the potency and therapeutic profiles. It is important to consider that this review is only based on small non-controlled clinical trials (not head-to-head comparison), and any switch of the products should be based on the approved product information. More importantly, physicians could put patients at risk if they incorrectly establish the dose equivalence.

Despite the difficulties related to the biologic units, the most informative comparisons of BoNT-A containing products have been made in clinical studies.

## 3. Dose Equivalence

Although there are some difficulties establishing the comparative potencies, the equivalence ratio of the dose should be established. The reasons for identifying a conversion factor are medical (*i.e.*, patients may need to switch to another formulation) as well as economical (an incorrect conversion factor may negatively impact the real cost of treatment), since each BoNT-A formulation contains different amounts of the 150-kD toxin (and NAPs)/LD50 unit (Table 1).

INCO was shown to be as effective as ONA with a comparable adverse event profile when a clinical conversion ratio of 1:1 or 1:1.2 was used [7,8,9,10,11]. Clinical data are consistent with preclinical comparability data [2,12]. Thus, both clinical and preclinical analyses have demonstrated a clinical conversion ratio between ONA and INCO very close to 1:1.

In contrast, the conversion ratio between ONA (or INCO, consequently) and ABO is hotly debated. Even if the most commonly quoted conversion ratios are 1:3 or 1:4 [13], they ranged from 1:1 [14] to as high as 1:11 [15]. This wide conversion ratio range reflects real-life clinical practice; the treating physician determines the number of muscles to be treated and the empiric dose based on each patient’s disease condition, their impairment pattern, and treatment objectives.

Although the various BoNT products differ with regard to NAP composition, the toxins ultimately inhibit acetylcholine release. Since the active toxin content is established for each product, a conversion rate should be defined. More precise estimation of conversion ratios should also ensure the development of comparable clinical data on the efficacy and safety of currently available BoNT-A formulations since they have qualitatively and quantitatively similar clinical efficacies and side effects at equipotent doses.

A large number of studies have reported an ONA:ABO conversion factor of 1:3. In order to establish an appropriate conversion factor, we evaluated the efficacy and safety in studies using a conversion factor >1:3.

All relevant studies using an ONA:ABO conversion factor ≤1:3 reported clinical equivalence (Table 2) [16,17,18,19,20]. Moreover, when the conversion factor is close to 1:3, ABO shows higher efficacy [21,22,23], indicating that the conversion factor is rather lower than equal to 1:3. Even more interesting are the studies where the conversion ratio was higher than 1:3. In these studies, it is clear that ABO showed higher efficacy and longer duration of action compared to ONA, but with more adverse events, by demonstrating that the conversion ratio >1:3 determines an overdose of ABO [24,25,26,27,28].

These clinical data are consistent with preclinical data where a conversion ratio for ONA/ABO of 1:3 or lower has been found [31,39].

An ONA:ABO conversion ratio of 1:4 or higher was adopted empirically in clinical practice before the availability of substantial clinical data. The current data suggest that a conversion ratio of 1:3—or even lower—could be appropriate for treating spasticity, cervical dystonia, and blepharospasm or hemifacial spasm. A higher conversion ratio may lead to an excessive ABO dose and the potential for an increased incidence of adverse events or under-dosing when switching ABO to ONA.

## 4. Toxin Spread

Toxin spread (also called diffusion and field of effect) describes the toxin’s effect on areas away from the injection site. Toxin spread to contiguous areas could be undesirable because it may increase the risk of adverse effects. For example, spread from injections in the cervical or craniofacial musculature may induce diplopia, dysarthria, or dysphagia.

Although uncommon, distant spread can cause unintended neuromuscular blockade at anatomic structures remote to the injection site. For example, systemic botulism symptoms such as dysphagia can occur when the toxin is injected at a distant site (e.g., lower extremities for spasticity). The potential risk for adverse effects due to toxin spread is described in the labeling for each BoNT-A product [40,41,42]. The mechanism of this phenomenon remains unknown.

Any potential differences in toxin spread characteristics and field of denervation among the BoNT products would be clinically relevant. Differences in the potential for contiguous spread among the BoNT products have been studied, but there is no evidence to date that differentiates the various products. There is a belief that diffusion of neurotoxin into adjacent tissue is slower with the high molecular weight complex compared with the lower molecular weight or free neurotoxin [12]. Theoretically, ONA with the highest complex size of 900 kD should be less diffusible, whereas INCO containing only the 150-kD neurotoxin (without NAPs) should be the most diffusible with a higher rate of side effects related to toxin spread, but this has not been proven. Progenitor toxin size may be irrelevant with regard to toxin diffusion because all BoNT progenitor complexes immediately dissociate following injection into a physiologic environment [43]. In fact, dissociation probably occurs in the vial on reconstitution with normal saline [44]. This is consistent with data from an animal model, in which there were no significant differences in the field of effect among ABO, INCO, and ONA [45]. Several factors other than the pharmaceutical preparation, such as clinical dose, solution concentration, injection technique, type of target site, location of injection within the muscle, level of muscle hyperactivity, depth of injection, and use of post-injection massage are thought to affect the potential for contiguous spread [46,47,48].

## 5. Immunogenicity

An important reason for secondary treatment failure of any therapeutic protein is its neutralization [49]. Antibodies that block its pharmacological effects are termed neutralizing or blocking antibodies and are addressed against the active toxin. The clinical effect may wane gradually, eventually leading to complete treatment failure.

In a study of 27 patients with complete treatment failure due to neutralizing antibodies, the majority (81%) of patients had previously experienced partial antibody-induced treatment failure [50]. Most patients in this study developed complete treatment failure within 40 months of starting botulinum toxin treatment. However, a more recent study reported a high mean clinical benefit, based on a 0–3 scale (0 = no effect, 1 = slight, 2 = moderate, and 3 = marked improvement) similar for ABO (2.5 ± 0.3) and ONA (2.2 ± 0.4), and <2% of the patients developed neutralizing antibodies [22]. In another investigation, BoNT-A antibodies were not detected in any of the study patients [51].

The debate regarding immunogenicity includes the role of the non-toxic clostridia proteins, collectively referred to as complexing proteins or neurotoxin-associated proteins (NAPs). Under physiological pH conditions, the complexing proteins dissociate almost completely from the neurotoxin following constitution with saline and even before injection into the target tissue [44,52]. Therefore, complexing proteins are not expected to modify clinical outcomes, and specific antibodies generated against the complexing proteins are termed non-neutralizing and should not affect the secondary response. However, it has been argued that complexing proteins may increase the bacterial protein load and can potentially increase the immunogenic risk of neutralizing antibody formation [53]. Although several studies have been performed, there are no clear demonstrations that NAPs may modify the immunogenicity of the active toxin [54,55,56]. However, these studies revealed that the toxoid complex is more immunogenic than the purified neurotoxin. This could be relevant considering that cross-reactivity may occur between the toxoid and toxin. The immunogenicity of toxoid components is of relevance since toxoid components (*i.e.*, inactive neurotoxin, albeit not through formaldehyde inactivation) are in some commercial botulinum toxin products. ONA is unique among the BoNT-A formulations in that it is vacuum-dried by a process involving sodium chloride, which may have a detrimental effect on neurotoxin activity [57] and may be responsible for its higher toxoid (inactive neurotoxin) content Despite the considerations mentioned above, the risk of immunogenicity of ONA is very low in clinical practice as reported by a large data review [58].

## 6. Pharmacoeconomic Considerations

The costs of treating patients with upper motor neuron lesions and spasticity are estimated to be four times greater than for those without spasticity or dystonia [59]. Spasticity-related costs generally include the costs of conventional treatment including hospitalization, rehabilitative therapy, and pharmacotherapy.

The use of BoNT-A is considered an effective [60,61] and potentially cost-effective [62,63,64] antispastic pharmaceutical treatment as an adjunct to conventional treatment. As mentioned above, there are variations in the composition, properties, and cost of the three BoNT-As being used to manage spasticity.

Some studies have assessed the cost per patient of the three BoNT-As. Roze *et al.* [65] compared the cost per patient per injection for Dysport and Botox in 19 countries. The recommended dosages in the summary of product characteristics are 1000 U and 300 U per patient, respectively. The cost per patient per injection for upper limb spasticity was less for Dysport than for Botox in 18 (95%) of the 19 countries (mean 17% less across countries). The difference was 20% or higher in nearly half (47%) of the countries. Sensitivity analyses considering available “real-world” dosing showed consistent results, with Dysport being less costly than Botox in all 19 countries. The authors concluded that substantial savings could be realized by using Dysport to treat upper limb spasticity.

Abogunrin *et al.* [66] recently developed a budget impact model to assess the effect of changing market shares of different BoNT-A formulations (ABO, ONA, and INCO) and the best supportive care from the UK payer perspective. The results demonstrate that BoNT-A treatment costs less than the best supportive care per patient per year, although treating a patient with ONA (£20,861) and INCO (£20,717) cost more per patient annually than ABO (£19,800). The authors concluded that increased use of ABO for upper limb spasticity could potentially reduce the total upper limb spasticity cost for the health system and society.

With regard to dystonia, botulinum toxin is considered an expensive drug with good effects [11]. From a societal perspective, the costs may well be worth the regained quality of life. However, the available literature concerning costs, health-related quality of life, and labor participation is very limited. Two recent reports evaluated the comparative costs of botulinum toxin type A. One evaluated a cost-utility analysis of BONT-A products for the treatment of cervical dystonia [67]. All three botulinum toxin type A agents were cost-effective at a willingness-to-pay threshold of $100,000 per quality-adjusted life year (QALY). Xeomin was the most cost-effective with a cost-effectiveness ratio of $27,548 per QALY. Dysport had the second-lowest cost-effectiveness ratio ($36,678), followed by Botox ($49,337). On the other hand, in a retrospective study [68] including patients with blepharospasm (*n* = 19), cervical dystonia (*n* = 122), hemifacial spasm (*n* = 91), and segmental/generalized dystonia (*n* = 19), switching from ABO to INCO reduced treatment costs. Although no exact dose conversion is available, both studies reported a 4:1 dosing equivalency between Botox/Xeomin and Dysport. Finally, in another recent study, INCO administered at flexible treatment intervals determined by the needs of the patient was found to be a cost-effective treatment option when compared to the administration of ONA in the Australian health care system [69].

## 7. Conclusions

BoNT therapy is considered a first-line therapy for a number of overactive muscle conditions such as upper and lower limb spasticity, focal dystonias such as cervical dystonia and blepharospasm, and hemifacial spasm. It has superior efficacy and safety compared with standard medical therapies (e.g., antispasmodics, muscle relaxants, neurolytics) or surgical interventions. The pharmacologic potency, high specificity, and long duration of BoNT-A make these toxins remarkably effective therapeutic agents for managing disorders characterized by muscle hyperactivity. There are three BoNT-A containing products on the market: Botox, Dysport, and Xeomin. All three preparations have similar mechanisms of action. The major difference between them relates to potency and the presence or absence of complexing proteins; therefore, the dose equivalence is important in clinical practice. ONA and INCO have comparable efficacies with a 1:1 conversion ratio and have demonstrated therapeutic equivalence in different indications including cervical dystonia and blepharospasm. An ONA to ABO conversion ratio ≤1:3 should be considered the most appropriate. Immunogenicity is another factor that may affect clinical efficacy after repeated injections. Finally, whereas the three BONT-A have similar efficacies when dosed properly, ABO has a better cost-efficacy profile.

In conclusion, it seems important to reiterate that comparability between the various BONT-A preparations was determined with indirect methods and since there is no standardized potency test among all three products, clinical trials are needed to establish the exact conversion ratio.

## Figures and Tables

**Table 1:** Botulinum toxin products and protein content/100 units [5,6].

Nonproprietary Name	150-kD Protein Content (ng)	Total Protein (150 kD and NAP) Content (ng)	Dose Equivalent Units
Onabotulinumtoxin A	0.73	5.00	1
Incobotulinumtoxin A	0.44	0.44	1
Abobotulinumtoxin A	0.65	0.87	2–3

NAP = nontoxic accessory proteins.

**Table 2:** Studies using an ONA:ABO conversion factor ≤1:3.

Authors	Study	Authors‘ Conclusions
Marion *et al.*, 1995 [16]	Open study of 74 pts, 37 with idiopathic blepharospasm and 37 with hemifacial spasm switched from ONA to ABO 1:3 ratio	Correct ONA:ABO conversion ratio is 1:3
Whurr *et al.*, 1995 [17]	Open study 16 pts with spasmodic dysphonia	Correct conversion ratio ONA:ABO is 1:3
Sampaio *et al.*, 1997 [24]	RCT 91 pts with blepharospasm and hemifacial conversion ratio ONA:ABO 1:4	ABO groups, in the conditions applied in the included trials, tend to have a higher efficacy, longer duration of action, and higher frequency of adverse reactions; A 1:4 ONA:ABO ratio is too high
Odergren *et al.*, 1998 [19]	RCT of 73 patients with CD ABO (*n* = 38) *vs.* ONA (*n* = 35) Conversion ratio 3:1	Efficacy and tolerability equivalent with an ABO:ONA ratio of 3:1
Tidswell and King, 2001 [26]	Open study 35 pts with CD switched from ONA to ABO conversion ratio 1:5	1:5 is too high; proposed 1:3. The authors report with insufficient efficacy and duration of action with ONA, suggesting that an ONA:ABO conversion ratio of 1:3 is more appropriate
Ranoux *et al.*, 2002 [27]	RCT, cross-over 54 pts with CD Conversion ratio ABO:ONA 3:1 or 4:1	Both with a ratio 3:1 and 4:1, they observed a higher and longer clinical efficacy of ABO *vs.* ONA with a higher risk of side effects; This suggests that the 3:1 conversion ratio is more appropriate
Poewe, 2002 [29]	RCT 54 pts with CD Conversion ratio ABO:ONA 3:1 or 4:1	The author comment on Ranoux′s paper confirming its conclusions: the ABO:ONA conversion ratio should not be >3:1
Sampaio *et al.*, 2004 [30]	*Systematic review* Blepharospasm CD/hemifacial spasm	The ABO:ONA 4:1 ratio is clearly too high, and even with a ratio of 3:1, ABO continues to have a longer duration of action
Wohlfarth *et al.*, 2008 [21]	79 healthy volunteers	ABO:ONA ratio 3:1 too high Equivalence ratio of 1.57:1 (95% CI: 0.77–3.2) To investigate the 2:1 ratio
Van den Berg *et al.*, 1998 [31]	Open study 10 pts with DC 10 pts with blepharospasm switched to ABO from ONA Conversion ratio 2.36:1	Dose equivalence ABO:ONA = 2.36:1
Rosales *et al.*, 2006 [32]	Review of preclinical and clinical studies	Appropriate conversion ratio ABO:ONA equal to 2.5–3:1 or lower
Wohlfarth *et al.*, 2009 [33]	Review of clinical studies	Dose equivalence ABO:ONA 2–2.5:1. Conversion ratios ≥4:1 should be considered overdosed for ABO
Shin *et al.*, 2009 [20]	Open study of 48 pts with blepharospasm switched to ABO from ONA; conversion ratio 2.5:1	Clinical and safety equivalence
Mohammadi *et al.*, (2009) [22]	Retrospective study 137 patients with spasticity, conversion ratio ABO:ONA 2 to 3:1	Clinical and safety equivalence
Kollewe *et al.*, 2010 [18]	97 pts with hemifacial spasm treated with ABO or ONA	Clinical and safety equivalence at conversion ratio of 2.56:1
Karen-Capelovitch *et al.*, 2010 [34]	16 pts with cerebral spastic palsy treated with ONA 12 U/kg or ABO 30 U/kg (ratio 1:2.5)	Clinical equivalence
Rystedt *et al.*, 2012 [23]	Retrospective study of 75 pts with CD	1.7:1 is the more appropriate ABO:ONA conversion ratio
Brockmann *et al.*, 2012 [35]	Retrospective study of 51 pts with Cervical CD	Dose equivalence ABO:ONA 3:1; Conversion ratios ≥ of 4:1 or superior should be considered overdosed for ABO
Kollewe *et al.*, 2014 [36]	Retrospective study of 288 patients with blepharospasm Conversion ratio ONA:ABO 1:2.3	No significant differences with regard to safety or efficacy
Rystedt *et al.*, 2015 [37]	RCT compares ONA and ABO in two different dose conversion ratios (1:3 and 1:1.7) when diluted to the same concentration (100 U/mL) for 46 patients with CD	No significant differences were seen between ONA and ABO (1:1.7); At week 12, a statistically significant difference in efficacy between ONA and ABO (1:3) was observed, suggesting a shorter duration of effect for ONA when this ratio (low dose) was used
Yun, 2015 [38]	103 patients with CD in a two-period crossover RCT	With regard to safety and efficacy, ABO was not inferior to ONA in patients with CD at a conversion factor of 2.5:1

ABO = abobotulinumtoxinA, CD = cervical dystonia; CI = confidence interval; ONA = onabotulinumtoxin A; RCT = randomized controlled trial.

## References

[1] Aoki K.R., Guyer B. (2001). Botulinum toxin type A and other botulinum toxin serotypes: A comparative review of biochemical and pharmacological actions. Eur. J. Neurol..

[2] Dressler D., Benecke R. (2007). Pharmacology of therapeutic botulinum toxin preparations. Disabil. Rehabil..

[3] Sesardic D., Leung T., Gaines-Das R. (2003). Role for standards in assays of botulinum toxins: International collaborative study of three preparations of botulinum type A toxin. Biologicals.

[4] McLellan K., Das R.E., Ekong T.A., Sesardic D. (1996). Therapeutic botulinum type A toxin: Factors affecting potency. Toxicon.

[5] Chen J.J., Dashtipour K. (2013). Abo-, Inco-, Ona-, and Rima-Botulinum toxins in clinical therapy: A primer. Pharmacotherapy.

[6] Frevert J. (2015). Pharmaceutical, biological, and clinical properties of botulinum neurotoxin type A products. Drugs R & D.

[7] Benecke R., Jost W.H., Kanovsky P., Ruzicka E., Comes G., Grafe S. (2005). A new botulinum toxin type A free of complexing proteins for treatment of cervical dystonia. Neurology.

[8] Roggenkamper P., Jost W.H., Bihari K., Comes G., Grafe S., NT 201 blepharospasm study team (2006). Efficacy and safety of a new botulinum toxin type A free of complexing proteins in the treatment of blepharospasm. J. Neural Transm..

[9] Jost W.H., Kohl A., Brinkmann S., Comes G. (2005). Efficacy and tolerability of a botulinum toxin type A free of complexing proteins (NT 201) compared with commercially available botulinum toxin type A (BOTOX) in healthy volunteers. J. Neural Transm..

[10] Park J., Lee M.S., Harrison A.R. (2011). Profile of Xeomin^®^ (incobotulinumtoxinA) for the treatment of blepharospasm. Clin. Ophtalmol..

[11] Zoons E., Dijkgraaf M.G.W., Dijk J.M., van Schaik I.N., Tijssen M.A. (2012). Botulinum toxin as treatment for focal dystonia: A systematic review of the pharmaco-therapeutic and pharmaco-economic value. J. Neurol..

[12] Dressler D., Mander G., Fink K. (2012). Measuring the potency labelling of onabotulinumtoxinA (Botox^®^) and incobotulinumtoxinA (Xeomin^®^) in an LD50 assay. J. Neural Transm..

[13] Aoki K.R., Ranoux D., Wissel J. (2006). Using translational medicine to understand clinical differences between botulinum toxin formulations. Eur. J. Neurol..

[14] Wohlfarth K., Goschel H., Frevert J., Dengler R., Bigalke H. (1997). Botulinum A toxins: Units *versus* units. Arch. Pharmacol..

[15] Marchetti A., Magar R., Findley L., Larsen J.P., Pirtosek Z., Ruzicka E., Jech R., Sławek J., Ahmed F. (2005). Retrospective evaluation of the dose of Dysport and BOTOX in the management of cervical dystonia and blepharospasm: The REAL DOSE study. Mov. Disord..

[16] Marion M.H., Sheehy M., Sangla S., Soulayrol S. (1995). Dose standardisation of botulinum toxin. J. Neurol. Neurosurgery Psychiatry.

[17] Whurr R., Brookes G., Barnes C. (1995). Comparison of dosage effects between the American and British botulinum toxin A product in the treatment of spasmodic dysphonia. Mov. Disord..

[18] Kollewe K., Mohammadi B., Dengler R., Dressler D. (2010). Hemifacial spasm and reinnervation synkinesias: Long-term treatment with either Botox or Dysport. J. Neural Transm..

[19] Odergren T., Hjaltason H., Kaakkola S., Solders G., Hanko J., Fehling C., Marttila R.J., Lundh H., Gedin S., Westergren I. (1998). A double-blind, randomised, parallel group study to investigate the dose equivalence of Dysport and Botox in the treatment of cervical dystonia. J. Neurol. Neurosurgery Psychiatry.

[20] Shin J.H., Jeon C., Woo K.I., Kim Y.D. (2009). Clinical comparability of Dysport and Botox in essential blepharospasm. J. Korean Ophthalmol. Soc..

[21] Wohlfarth K., Schwandt I., Wegner F., Jürgens T., Gelbrich G., Wagner A., Bogdahn U., Schulte-Mattler W. (2008). Biological activity of two botulinum toxin type A complexes (Dysport^®^ and Botox^®^) in volunteers: A double-blind, randomized, dose-ranging study. J. Neurol..

[22] Mohammadi B., Buhr N., Bigalke H., Krampfl K., Dengler R., Kollewe K. (2009). A long-term follow up of botulinum toxin A in cervical dystonia. Neurol. Res..

[23] Rystedt A., Nyholom D., Naver H. (2012). Clinical experience of dose conversion ratios between toxin products in the treatment of cervical dystonia. Clin. Neuropharmacol..

[24] Sampaio C., Ferreira J.J., Simões F., Rosas M.J., Magalhães M., Correia A.P., Bastos-Lima A., Martins R., Castro-Caldas A. (1997). Dysport: A single-blind, randomized parallel study to determine whether any differences can be detected in the efficacy and tolerability of two formulations of botulinum toxin type A—Dysport and Botox, assuming a ratio of 4:1. Mov. Disord..

[25] Nussgens Z. (1997). Comparison of two botulinum toxin preparations in the treatment of essential blepharospasm. Graefe′s Arch. Clin. Exp. Ophthalmol..

[26] Tidswell P., King M. (2001). Comparison of Two Botulinum Toxin Type-A Preparations in the Treatment of Dystonias.

[27] Ranoux D., Gury C., Fondarai J., Mas J.L., Zuber M. (2002). Respective potencies of Botox and Dysport: A double-blind, randomised, crossover study in cervical dystonia. J. Neurol. Neurosurgery Psychiatry.

[28] Bentivoglio A.R., Ialongo T., Bove F., de Nigris F., Fasano A. (2012). Retrospective evaluation of the dose equivalence of Botox and Dysport in the management of blepharospasm and hemifacial spasm: A novel paradigm for a never ending story. Neurol. Sci..

[29] Poewe W. (2002). Respective potencies of Botox and Dysport: A double blind, randomised, crossover study incervical dystonia. J. Neurol. Neurosurgery Psychiatry.

[30] Sampaio C., Costa J., Ferreira J.J. (2004). Clinical comparability of marketed formulations of botulinum toxin. Mov. Disord..

[31] Van den Bergh P.Y.K., Lison D.F. (1998). Dose standardization of botulinum toxin. Adv. Neurol..

[32] Rosales R.L., Bigalke H., Dressler D. (2006). Pharmacology of botulinum toxin: Differences between type A preparations. Eur. J. Neurol..

[33] Wohlfarth K., Sycha T., Ranoux D., Naver H., Caird D. (2009). Dose equivalence of two commercial preparations of botulinum neurotoxin type A: Time for a reassessment?. Curr. Med. Res. Opin..

[34] Keren-Capelovitch T., Jarus T., Fattal-Valevski A. (2010). Upper extremity function and occupational performance in children with spastic cerebral palsy following lower extremity botulinum toxin injections. J. Child Neurol..

[35] Brockmann K., Schweitzer K., Beck G., Wächter T. (2012). Comparison of different preparations of botulinumtoxinA in the treatment of cervical dystonia. Neurol. Asia.

[36] Kollewe K., Mohammadi B., Köhler S., Pickenbrock H., Dengler R., Dressler D. (2015). Blepharospasm: Long-term treatment with either Botox®, Xeomin® or Dysport®. J. Neural Transm..

[37] Rystedt A., Zetterberg L., Burman J., Nyholm D., Johansson A. (2015). A comparison of Botox 100 U/mL and Dysport 100 U/mL using dose conversion ratio 1:3 and 1:1.7 in the treatment of cervical dystonia: A double-Blind, randomized, crossover trial. Clin. Neuropharmacol..

[38] Yun J.Y., Kim J.W., Kim H.T., Chung S.J., Kim J.M., Cho J.W., Lee J.Y., Lee H.N., You S., Oh E. (2015). Dysport and Botox at a ratio of 2.5:1 units in cervical dystonia: A double-blind, randomized study. Mov. Disord..

[39] Hambleton P., Pickett A.M. (1994). Potency equivalence of botulinum toxin preparations. J. R. Soc. Med..

[40] Highlights of prescribing information. http://www.allergan.com/assets/pdf/botox_pi.pdf.

[41] Dysport 300 units, Dysport 500 units—Summary of Product. https://www.medicines.org.uk/emc/medicine/870/SPC/Dysport+300+units,+Dysport+500+units/.

[42] Full prescribing information—Xeomin. http://www.xeomin.com/consumers/pdf/xeomin-full-prescribing-information.pdf.

[43] Wagman J., Bateman J.B. (1953). Botulinum type A toxin: Properties of toxic dissociation product. Arch. Biochem. Biophys..

[44] Eisele K.H., Fink K., Vey M., Taylor H.V. (2011). Studies on the dissociation of botulinum neurotoxin type A complexes. Toxicon.

[45] Carli L., Montecucco C., Rossetto O. (2009). Assay of diffusion of different botulinum neurotoxin type A formulations injected in the mouse leg. Muscle Nerve.

[46] Brodsky M.A., Swope D.M., Grimes D. (2012). Diffusion of botulinum toxins. Tremor Other Hyperkinet. Mov..

[47] Roche N., Schnitzler A., Genet F.F., Durand M.C., Bensmail D. (2008). Undesirable distant effects following botulinum toxin type A injection. Clin. Neuropharmacol..

[48] Pickett A. (2009). Dysport: Pharmacological properties and factors that influence toxin action. Toxicon.

[49] Kromminga A., Schellekens H. (2005). Antibodies against erythropoietin and other protein based therapeutics: An overview. Ann. N.Y. Acad. Sci. USA.

[50] Dressler D. (2002). Clinical features of antibody-induced complete secondary failure of botulinum toxin therapy. Eur. Neurol..

[51] Bakheit A.M., Liptrot A., Newton R., Pickett A.M. (2012). The effect of total cumulative dose, number of treatment cycles, interval between injections, and length of treatment on the frequency of occurrence of antibodies to botulinum toxin type A in the treatment of muscle spasticity. Int. J. Rehabil. Res..

[52] Benecke R. (2012). Clinical relevance of botulinum toxin immunogenicity. Biodrugs.

[53] Kukreja R., Chang T.-W., Cai S., Lindo P., Riding S., Zhou Y., Ravichandran E., Singh B.R. (2009). Immunological characterization of the subunits of type A botulinum neurotoxin and different components of its associated proteins. Toxicon.

[54] Atassi M.Z. (2006). On the enhancement of anti-neurotoxin antibody production by subcomponents HA1 and HA3b of Clostridium botulinum type B 16S toxin haemagglutinin. Microbiology.

[55] Atassi M.Z. (2004). Basic immunological aspects of botulinum toxin therapy. Mov. Disord..

[56] Bigalke H., Jankovic J., Albanese A., Atassi M.Z., Dolly J.O., Hallett M., Mayer N.H. (2009). Properties of pharmaceutical products of botulinum neurotoxins. Botulinum Toxin: Therapeutic Clinical Practice and Science.

[57] Frevert J. (2010). Content of botulinum neurotoxin in Botox/Vistabel, Dysport/Azzalure, and Xeomin/Bocouture. Drugs R & D.

[58] Jankovic J., Esquenazi A., Fehlings D., Freitag F., Lang A., Naumann M. (2004). Evidence-based review of patient-reported outcomes with botulinum toxin type A. Clin. Neuropharmacol..

[59] Lundström E., Smits A., Borg J., Terént A. (2010). Four-fold increase in direct costs of stroke survivors with spasticity compared with stroke survivors without spasticity: The first year after the event. Stroke.

[60] Francis H.P., Wade D.T., Turner-Stokes L., Kingswell R.S., Dott C.S., Coxon E.A. (2004). Does reducing spasticity translate into functional benefit? An exploratory meta-analysis. J. Neurol. Neurosurgery Psychiatry.

[61] Lukban M.B., Rosales R.L., Dressler D. (2009). Effectiveness of botulinum toxin A for upper and lower limb spasticity in children with cerebral palsy: A summary of evidence. J. Neural Transm..

[62] Ward A., Roberts G., Warner J., Gillard S. (2005). Cost-effectiveness of botulinum toxin type A in the treatment of post-stroke spasticity. J. Rehabil. Med..

[63] Shaw L., Rodgers H., Price C., van Wijck F., Shackley P., Steen N., Barnes M., Ford G., Graham L., BoTULS Investigators (2010). BoTULS: A multicentre randomised controlled trial to evaluate the clinical effectiveness and cost-effectiveness of treating upper limb spasticity due to stroke with botulinum toxin type A. Health Technol. Assess..

[64] Doan Q.V., Gillard P., Brashear A., Halperin M., Hayward E., Varon S., Lu Z.J. (2013). Cost-effectiveness of onabotulinumtoxin A for the treatment of wrist and hand disability due to upper-limb post-stroke spasticity in Scotland. Eur. J. Neurol..

[65] Roze S., Kurth H., Hunt B., Valentine W., Marty R. (2012). Evaluation of the cost per patient per injection of botulinum toxin A in upper limb spasticity: Comparison of two preparations in 19 countries. Med. Devices.

[66] Abogunrin S., Brand S., Desai K., Dinet J., Gabriel S., Harrower T. (2015). Abobotulinumtoxin A in the management of cervical dystonia in the United Kingdom: A budget impact analysis. Clin. Econ. Outcomes Res..

[67] Kazerooni R., Broadhead C. (2015). Cost-utility analysis of botulinum toxin type A products for the treatment of cervical dystonia. Am. J. Health Syst. Pharm..

[68] Grosset D.G., Tyrrell E.G., Grosset K.A. (2015). Switch from abobotulinumtoxinA (Dysport^®^) to incobotulinumtoxinA (Xeomin^®^) botulinum toxin formulation: A review of 257 cases. J. Rehabil. Med..

[69] Tilden D., Guarnieri C., Jackson D. (2015). Cost-effectiveness of incobotulinumtoxin-A with flexible treatment intervals compared to onabotulinumtoxin-A in the management of blepharospasm and cervical dystonia. Value Health.

